# Faceting–roughening transition of a Cu grain boundary under electron-beam irradiation at 300 keV

**DOI:** 10.1038/s41598-021-95091-7

**Published:** 2021-07-30

**Authors:** Sung Bo Lee, Heung Nam Han

**Affiliations:** Department of Materials Science and Engineering and Research Institute of Advanced Materials (RIAM), Seoul National University, Seoul, 08826 South Korea

**Keywords:** Structural materials, Metals and alloys, Phase transitions and critical phenomena

## Abstract

In this study, we examined the beam-irradiation effect on the structural evolution of the grain boundary (GB) in a Cu bicrystal at room temperature using a C_s_-corrected, monochromated transmission electron microscope at an acceleration voltage of 300 keV. Faceting of the GB was observed at a low current density of the electron beam. With increasing current density, the GB became defaceted. The faceting–roughening transition was shown to be reversible, as the process was reversed upon decreasing the current density. The structural transition is explained by inelastic scattering effects by electron-beam irradiation.

## Introduction

Irradiation by electrons, neutrons or ions with high energies generate point defects on polycrystalline materials and these defects are absorbed and annihilated by grain boundaries (GBs)^1–4^. Further, in the case of electron irradiation, point defects were observed to induce GB migration. Sasaki and Saka^5^ examined GBs in an intermetallic compound (CuGa_2_) under electron-beam irradiation at 200–1000 keV using a transmission electron microscope (TEM) and observed an oscillation of the GBs. It was deduced that knock-on atomic displacement by electron-beam irradiation produced point defects, which enhanced the GB migration. However, intriguingly, at an acceleration voltage below the knock-on threshold a GB was observed to migrate^6^. Merkle and Thompson^6^ examined the GB in an Au bicrystal composed of [0 0 1] and [0 1 1] grains at a TEM operated at 400 keV at a current density of ~ 5 A cm^–2^. They observed that the GB was atomically sharp and migrated from the [0 0 1] grain into the [0 1 1] grain by the propagation of atomic steps. The threshold energy for knock-on displacement of Au is estimated to exceed 1000 keV^7^. Therefore, radiation-induced point defects are not expected to be generated at 400 keV. On the other hand, the threshold energy for sputtering of Au is ~ 270 keV^8^. Thus, the observation indicates that the observed GB migration and faceting occurred in the range of sputtering with a low probability of occurrence of Frenkel defects in the bulk of TEM specimen film. However, understanding of mechanisms behind GB migration and, further, GB structural transitions, such as faceting transition, under electron-beam irradiation with various acceleration voltages has been missing.

Here, we show that electron-beam irradiation in a TEM caused not only GB migration but also a GB structural transition. Electron-beam irradiation was performed in a TEM with an acceleration voltage of 300 keV. The Cu bicrystal was composed of two grains with the surface normal directions of [1 0 0] and [1 1 0]. At low current densities of the electron beam, the GB, which initially was defaceted, was faceted into two GB components and migrated from [1 0 0] to [1 1 0]. As the current density was further increased, the GB became defaceted. When the rough GB at the higher current density was subjected to a lower current density, it returned to a faceted structure composed of the two GB facets. These results indicate that the faceting–roughening GB transition under electron-beam irradiation was reversible. We attribute the GB structural transition and migration to inelastic scattering effects by electron-beam irradiation.

## Materials and microstructural characterization

**Figure 1:**
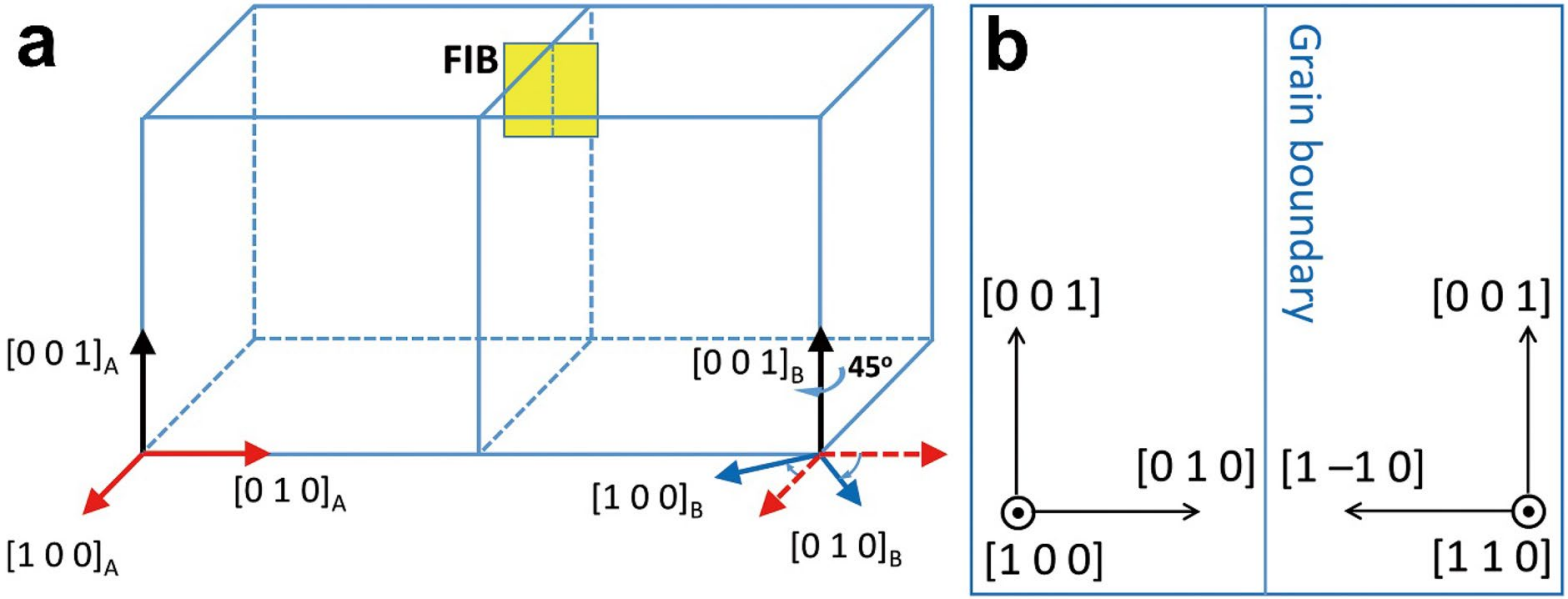
(**a**) Three-dimensional sketch showing crystallographic directions of the Cu bicrystal composed of two grains with a misorientation relationship of [0 0 1]/45°. (**b**) Illustration of crystallographic directions of an FIB specimen extracted from the GB area in the bicrystal.

The Cu bicrystal in this work was fabricated by pulling two Cu single-crystal seeds at a desired misorientation angle from the melt (Czochralski technique^9–11^) (MaTeck GmbH). The GB in the bicrystal was designed to have a misorientation relationship of [0 0 1]/45° with a pure tilt component and its plane was roughly parallel to the (0 1 0) and (1 −1 0) planes of two mating grains (Fig. 1). Cross-sectional specimens for high-resolution TEM (HRTEM) were prepared from the bicrystal by focused ion beam (FIB) [Nova 200 NanoLab (FEI) and Quanta 3D FEG (FEI)]. The FIB milling and polishing were done at 30 kV and at 5 kV, respectively. The Cu FIB lamella was composed of two grains with different surface normal directions of [1 0 0] and [1 1 0] (Fig. 1b). The specimen thickness was determined to be ~ 50 nm on average using the electron energy loss spectroscopy log ratio method.

Electron irradiation was performed in the TEM mode of a spherical-aberration corrected and monochromated TEM (Themis Z, Thermo Fisher Scientific, operated at 300 keV, column vacuum of ~ 6 × 10^–6^ Pa). The total beam current was changed by adjusting the gun lens and/or the second condenser (C2) aperture size. The beam current was measured on the fluorescent screen in vacuum with no specimen, which had been carefully calibrated using a Faraday cup. The beam current density at the specimen surface was determined by dividing the total beam current by the illumination area at the specimen surface. The illumination area was changed by spreading or contracting the beam by adjusting the C2 lens.

In the present study, irradiation was performed at a current of 4 nA with beam diameters of 50, 25, and 10 nm, which correspond to current densities of approximately 204, 815, and 5093 A cm^–2^, respectively. Regions irradiated at a diameter of 50 nm were observed in situ. However, those irradiated at diameters of 25 and 10 nm were observed ex situ at the same TEM to avoid beam damage to the CCD camera (Ceta 16 M). Ex situ observations were made at 4 nA with a beam diameter of 140 nm (~ 26 A cm^–2^) at magnifications of 460 k, 570 k, 720 k, 910 k, and 1.1 M. That is, imaging was performed after irradiation.

**Figure 2:**
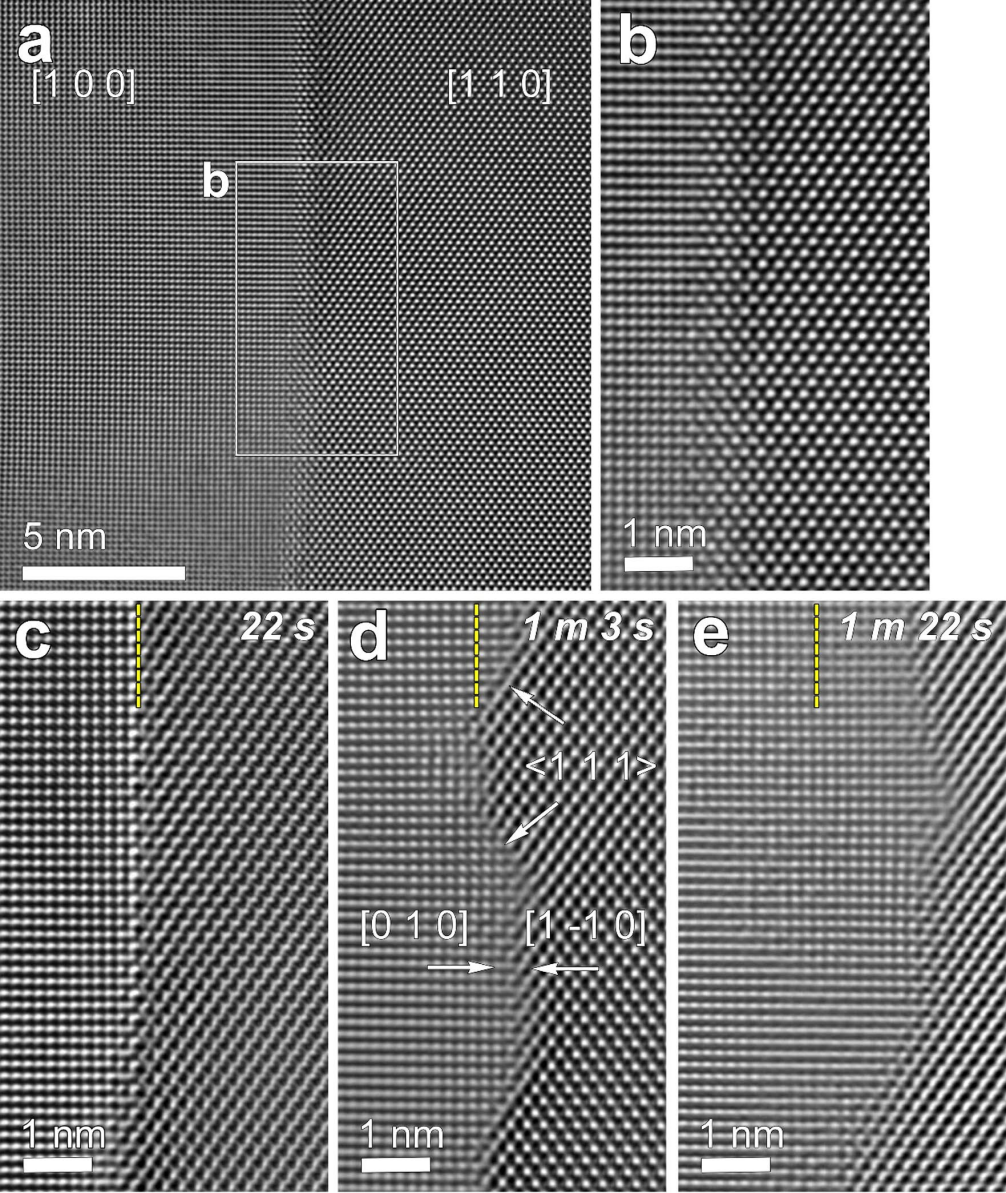
Sequential HRTEM images showing microstructural evolution of a GB region under beam irradiation with a beam diameter of 50 nm corresponding to a current density of ~ 204 A cm^–2^. The region shown in this figure was included in the irradiation diameter of 50 nm. (**a**) An initially rough GB. For reference, the irradiation diameter and corresponding current density are specified side-by-side. (**b**–**e**) Time evolution of the boxed region in (**a**) during irradiation obtained by in situ observation. The elapsed irradiation time in the condition is labeled at the upper right corner of each figure. (Minutes and seconds are abbreviated as m and s, respectively.) (**c**–**e**) The GB reveals atomically sharp (0 1 0)//(1 –1 0) and {1 1 1} facets and its migration into [1 1 0]. See text for details of the plane notations. The vertical yellow dashed lines in the figures indicate the initial position of the GB shown in (**c**).

**Figure 3:**
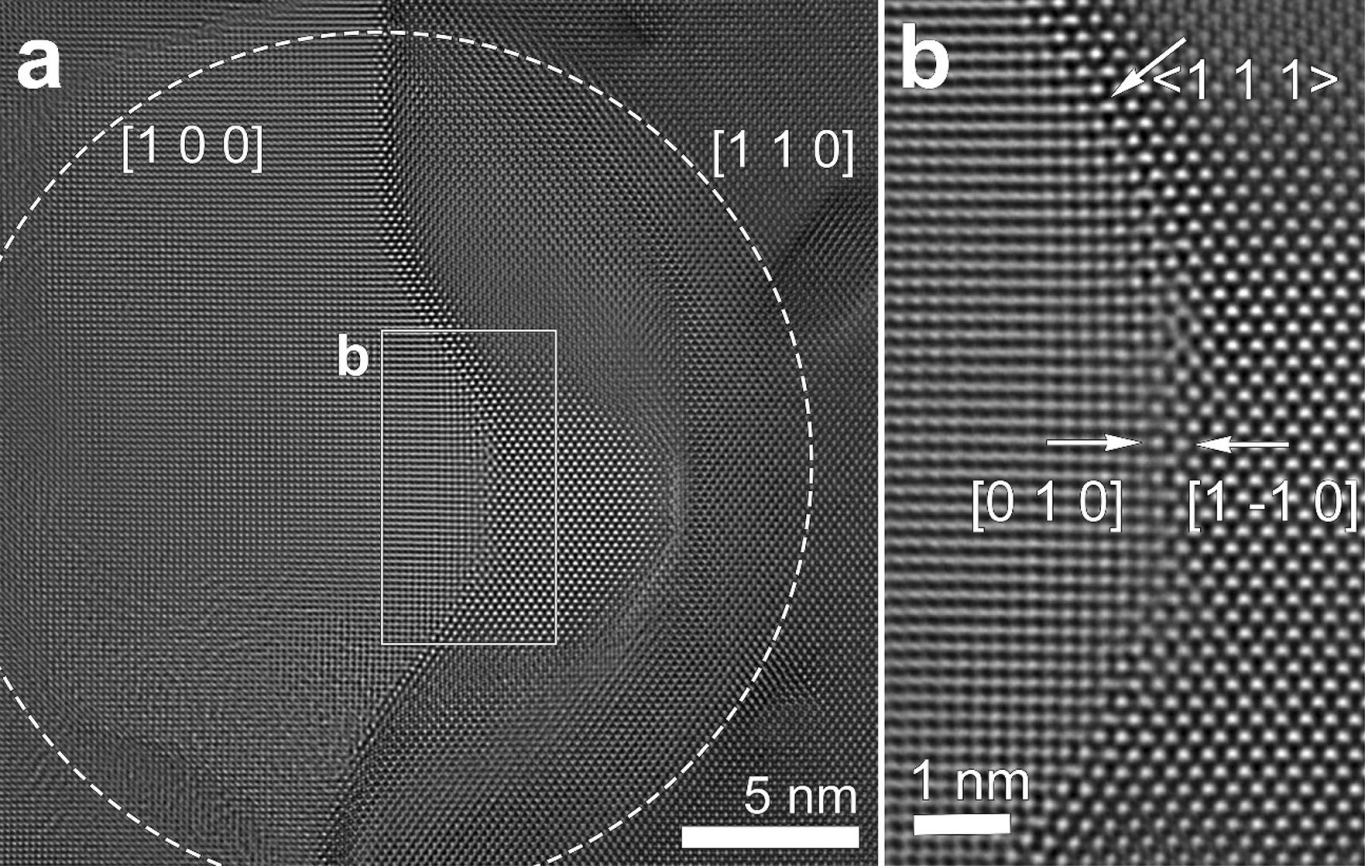
Sequential HRTEM images showing microstructural evolution of a GB region after beam irradiation with a beam diameter of 25 nm corresponding to a current density of ~ 815 A cm^–2^ for 3 min 38 s. (**a**) Low-magnification image showing GB migration from [1 0 0] to [1 1 0]. The irradiation diameter of 25 nm is marked by a dashed circle. (**b**) Enlarged image of the boxed region of (**a**), which shows that the region was faceted into atomically sharp (0 1 0)//(1 –1 0) and {1 1 1} components.

**Figure 4:**
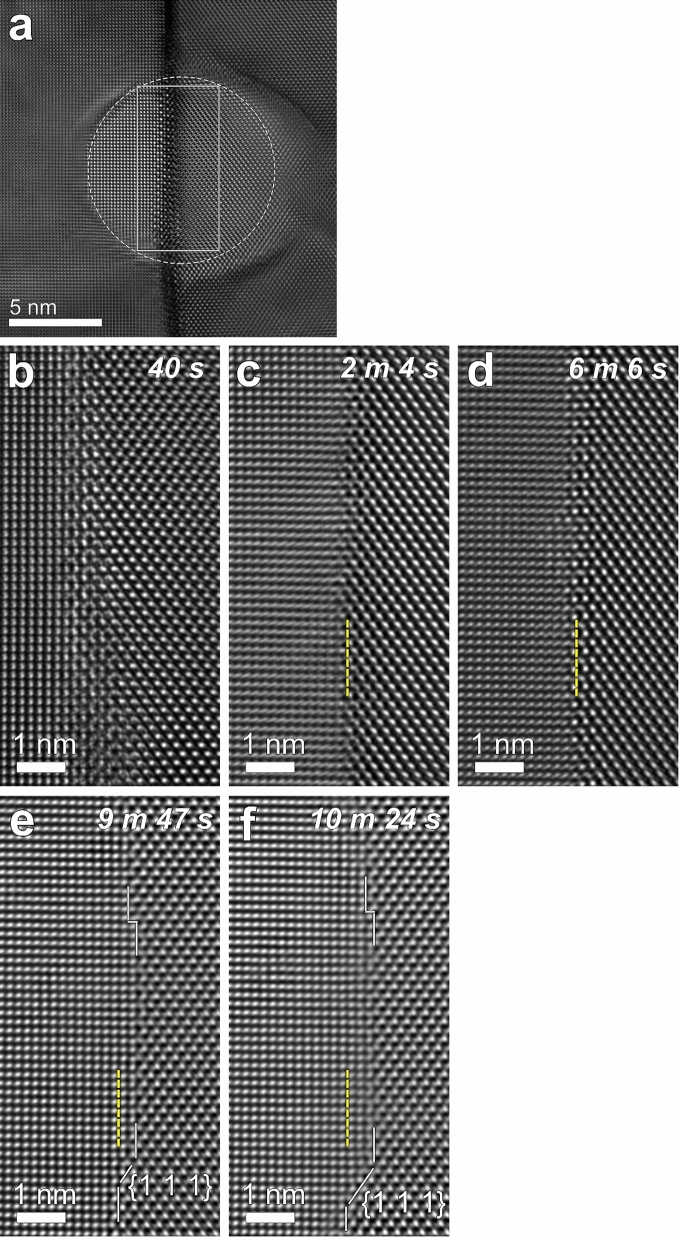
(**a**) Sequential HRTEM images showing microstructural evolution of GB region after irradiation with a beam diameter of 10 nm (~ 5093 A cm^–2^) for 1 min 30 s. The irradiation diameter of 10 nm is marked by a dashed circle. The GB region in (**a**) was subsequently irradiated at 50 nm (~ 204 A cm^–2^). (**b**–**f**) Time evolution of the boxed region in (**a**) at 50 nm, which was imaged during in situ observation, as for Fig. 2. The elapsed time at 50 nm is specified in the upper right corner of each figure. The vertical dashed lines in the figures indicate the initial position of the GB shown in (**c**).

**Figure 5:**
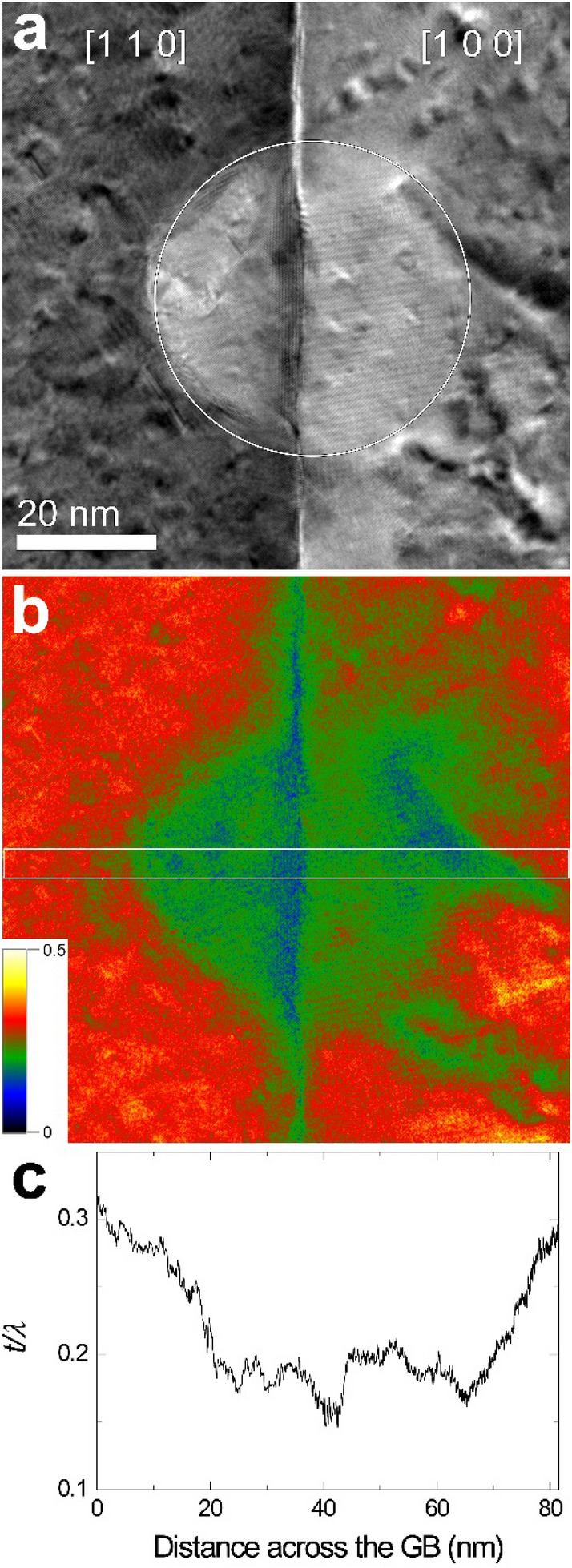
(**a**) EFTEM image (unfiltered) obtained from a beam irradiated area at 50 nm for 3 min 20 s and (**b**) a corresponding thickness map. (**c**) Line profile of relative thickness for the rectangular area shown in (**b**).

**Figure 6:**
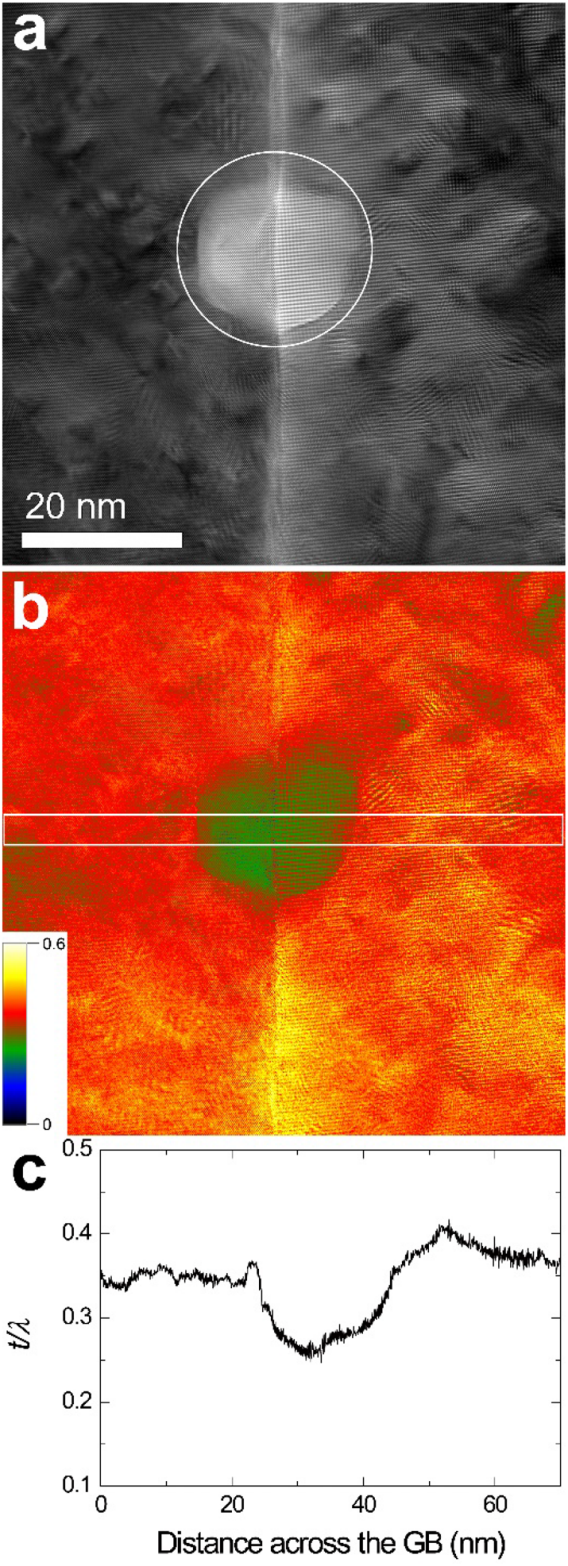
(**a**) EFTEM image (unfiltered) obtained from a beam irradiated area at 25 nm for 2 min 8 s and (**b**) a corresponding thickness map. (**c**) Line profile of relative thickness for the rectangular area shown in (**b**).

**Figure 7:**
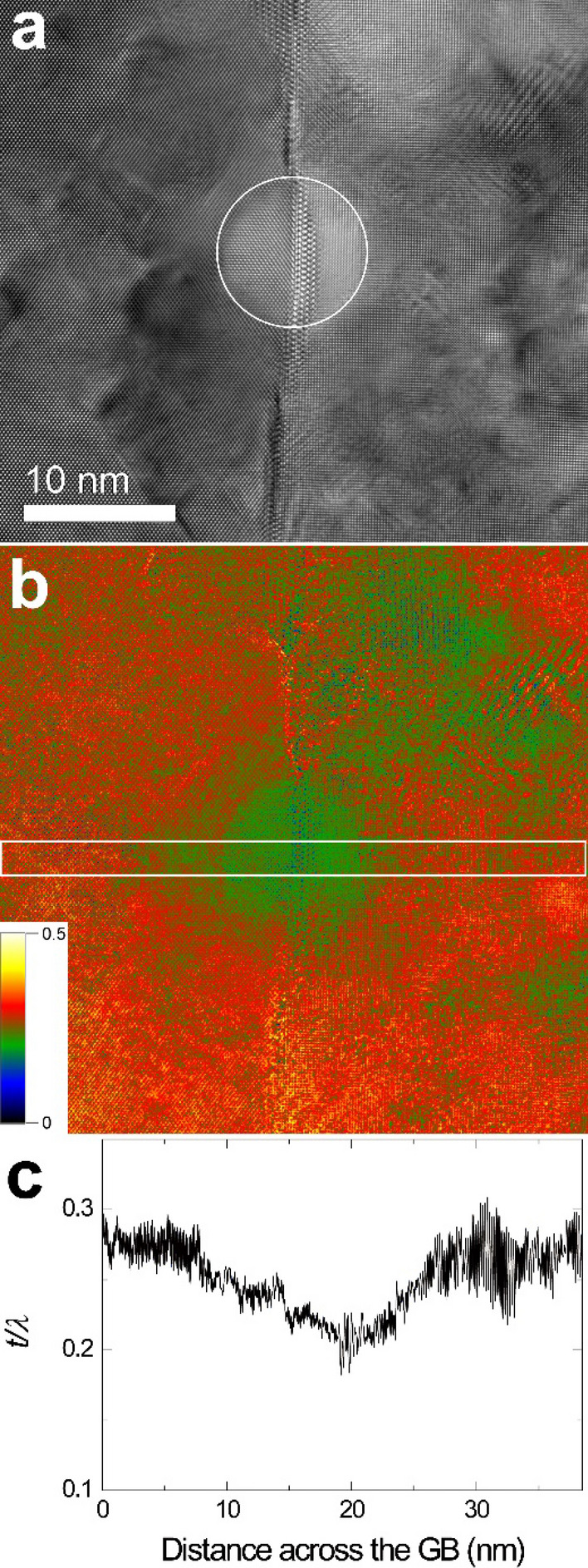
(**a**) EFTEM image (unfiltered) obtained from a beam irradiated area at 10 nm for 1 min 50 s and (**b**) a corresponding thickness map. (**c**) Line profile of relative thickness for the rectangular area shown in (**b**).

To confirm the thickness reduction due to sputtering, the thickness of the irradiated area and its surroundings was measured by energy-filtered TEM (EFTEM). First, other GB parts than the GBs used in Figs. 2, 3 and 4 were irradiated with diameters of 50, 25, and 10 nm, and then, thickness measurements were made. Relative thickness [absolute thickness (***t***)/mean free path (***λ***)] maps were acquired (Figs. 5, 6, 7).

## Experimental results

To illuminate the correlation between electron-beam irradiation and the GB structural change, the initial GB (Fig. 2a) was irradiated by focusing the electron beam of 4 nA over a diameter of 50 nm, corresponding to a current density of 204 A cm^–2^. For the irradiation at 50 nm, observations were made in situ, as noted in the Experimental section (Fig. 2). During irradiation at 50 nm, the initial GB gradually became atomically flat, revealing a facet parallel to the (0 1 0) and (1 −1 0) planes of the two mating grains [hereafter, designated as (0 1 0)//(1 –1 0)] and facets parallel to {1 1 1} planes of the [1 1 0] grain and nearly to {0 3 2} of the [1 0 0] grain [simply, designated as {1 1 1} facet] (Fig. 2c–e). It was observed to migrate from [1 0 0] to [1 1 0].

At a diameter of 25 nm (815 A cm^–2^) (Fig. 3), the GB was also faceted into two components, (0 1 0)//(1 –1 0) and {1 1 1}, and migrated from [1 0 0] to [1 1 0], as observed at the lower current density (Fig. 2).

Upon further increasing the current density to an irradiation diameter of 10 nm (5093 A cm^–2^) (Fig. 4a), no sharp facets were developed. In Fig. 4a, the initial GB region is outside the beam irradiated area marked by a dashed circle. The GB increased its width. As can be seen in the figure, both the initial GB and the migrated GB region were inclined with respect to the beam direction. Note that the GB part in the irradiated area became more inclined than the initial GB.

To explore whether the GB structural transition shown above (Figs. 2, 3) was reversible by changing the current density, the GB region irradiated at a diameter of 10 nm (Fig. 4a) was subsequently irradiated at a diameter of 50 nm, during which observations were made in situ (Fig. 4b–f). During the irradiation at 50 nm, the GB migrated from [1 0 0] to [1 1 0], as observed for the lower current densities (Figs. 2 and 3), which occurred by the lateral propagation of atomic-height steps on the (0 1 0)//(1 –1 0) facet with a height of one (2 0 0) interplanar spacing, as indicated by the guide lines in Fig. 4e,f. The step migration and bunching is considered to have resulted in the formation of {1 1 1} facets (Fig. 4e,f).

## Discussion

It might be assumed that the GB migration and faceting–roughening transition (Figs. 2, 3, 4) were induced by local beam heating by electron-beam irradiation. Specimen heating by electron-beam irradiation is calculated to be based on the premise that the heat generation in an incident beam of diameter *d* is balanced by heat loss due to radial conduction^8^. A calculation using the equation suggested by Egerton et al.^8^ shows that the temperature rise is negligible, lower than 0.01 K in the range of beam diameters from 50 to 10 nm, which is because of the high thermal conductivity *κ* of Cu (401 W m^–1^ K^–1^ for the bulk at 293 K).

The GB migration direction from [1 0 0] to [1 1 0] at the lower current densities at 50 and 25 nm (Figs. 2 and 3) is explained by the surface energy anisotropy. Because, for Cu, the [1 0 0] grain has a lower surface energy ($\upgamma$) than the [1 1 0] grain for Cu^12^, the surface energy difference ($\Delta\upgamma ={\gamma }_{110}-{\gamma }_{100}=2.237-2.166=$ 0.07 J m^−2^) between the two grains can provide a driving force for grain growth of the [1 0 0] grain, which is given by $2\Delta\upgamma /t$, where $t$ is the specimen thickness, which was determined to be ~ 50 nm on average. The relevant thickness results in a driving force of 2.8 MPa. At 10 nm (Fig. 4a), the GB was more inclined in the irradiated area, but did not show any distinct migration. This is probably because both grain surfaces were severely damaged by irradiation and the surface energy anisotropy would be reduced.

The minimum energy needed to displace an atom from its fcc lattice of Cu is 20 eV, corresponding to a threshold energy for knock-on displacement of 420 keV^8^. However, it is well accepted that the displacement of surface atoms (sputtering) occurs at voltages much lower than the knock-on threshold energy^8^. Egerton et al.^8^ regarded the sublimation energy per atom as the minimum energy required for sputtering. The sublimation energy per atom for Cu is 3.49 eV^13^, which corresponds to an incident energy of 93 keV. The acceleration voltage of the TEM used in the present study was between the sputtering threshold energy (93 keV) and the knock-on threshold energy for Cu (420 keV). Therefore, it is considered that the beam-irradiation condition was in a regime of sputtering.

As shown in Figs. 3 and 4, it can be seen that the thickness of the beam irradiation area became thinner, though it is not clear in Fig. 2. The thickness reduction could be confirmed by measuring the thickness of the beam irradiation area by energy-filtered TEM (Figs. 5, 6, 7). This indicates that the irradiation condition in the present study causes sputtering. As shown in Figs. 5, 6 and 7, the difference in thickness between the [1 0 0] and [1 1 0] grains after beam irradiation is not discernible, indicating that sputtering yield difference between the two grains was negligible. This result makes us deduce that the GB migration direction from [1 0 0] to [1 1 0] shown in Figs. 1, 2, 3 and 4 is not related to sputtering-related defects.

The possibility of the GB faceting–roughening transition under electron-beam irradiation is indirectly found in a recent study by Lee et al.^14^. In the study, at a fixed current density (0.768 A cm^–2^ at the acceleration voltage of 1250 keV using a high-voltage TEM), the structural changes of two types of the GBs were compared, which revealed that one GB was faceted and the other remained wavy. The phenomena were attributed to an increase in effective temperature^15,16^. The simple effective temperature criterion^15,16^ suggested that that irradiation of a material by high energy particles generates a high concentration of excess vacancies. The excess vacancies would be equivalent to a temperature increase from the actual temperature of experiment $\left({T}_{\mathrm{exp}}\right)$ to a certain effective temperature $\left({T}_{\mathrm{eff}}\right)$. When ${T}_{\mathrm{eff}}$ exceeds the thermal roughening transition temperature^17–21^ of an interface, the interface would undergo roughening^15,16^. This criterion^15,16^ simply applies to the case of the formation of Frenkel defects. For Au, the minimum energy required to displace an atom from its lattice, or briefly displacement energy, is 34 ± 7 eV^7,8^, which indicates that the threshold for knock-on displacement in the bulk exceeds 1000 keV. Therefore, at the acceleration voltage of 1250 keV, the examined specimens were subjected to a regime of knock-on damage, producing Frenkel defects^14^. There, the faceting transition in the Au GBs^15^ could be explained by the effective temperature criterion^15,16^.

However, in this work, the irradiation was done at 300 keV, where no formation of Frenkel defects is expected in the Cu bulk. The circumstance indicates that other processes are responsible for the phenomena. A hint about the possible mechanism behind the structural transition is noticed in inelastic scattering induced by electron-beam irradiation^22^. During in situ TEM tensile straining of Al and Au films at lower accelerating voltages (120 and 200 keV), Sarkar et al.^22^ recognized electron-beam induced effects. It was observed^22^ that electron-beam irradiation increased dislocation activation and thus resulted in stress relaxation in Al and Au films. The electrons inelastically scatter near lattice defects, such as dislocations, producing local lattice vibrations (phonons)^22^. The interaction of the phonons and dislocations increases the mobility of dislocations^22^. This scheme also applies to GBs (exactly, GB dislocation-disclinations^23,24^). The inelastic electron scattering at GBs is expected to accelerate GB mobility.

The phonon-induced GB mobility will increase with increasing the current density, because the frequency of inelastic scattering is expected to increase. At a higher current density, the GB mobility becomes higher, and therefore at a given driving force for migration, the GB will tend to be *kinetically* roughened, as shown in Fig. 4a. On the other hand, as the current density decreases, the GB mobility is likely to decrease and the GB will migrate, revealing faceted structure without being roughened, as shown in Figs. 2, 3, and 4b–f.

## Concluding remarks

To conclude, this work demonstrates faceting–roughening transition in a Cu grain boundary under electron-beam irradiation, which was reversible upon changing the current density. The present acceleration voltage of 300 keV corresponded to a sputtering regime under electron-beam irradiation. Therefore, the observed transition cannot be related to any formation of Frenkel defects, but is attributed to inelastic scattering effects by electron-beam irradiation. This work is of great significance in understanding the microstructural evolution under extreme radiation conditions, as in polycrystalline nuclear materials, by demonstrating that the GB structure can undergo structural transition under electron-beam irradiation.
